# Molecular Epidemiological Surveillance of HIV-1 Genotypes and Drug Resistance Profiles in Wuhan, Central China

**DOI:** 10.3390/v18010055

**Published:** 2025-12-30

**Authors:** Qiqi Zhang, Mingzhe Yan, Jingxin Huang, Yujie Liu, Hanji Wang, Sheng Feng, Zheng Dong, Dilihumaer Abulimiti, Youping Wang, Ke Liang, Yong Feng

**Affiliations:** 1 Department of Medical Microbiology, School of Basic Medical Sciences, Wuhan University, Wuhan 430071, China; 2 Wuhan Jinyintan Hospital, Tongji Medical College of Huazhong University of Science and Technology, Wuhan 430023, China; 3 Department of Infectious Diseases, Zhongnan Hospital of Wuhan University, Wuhan 430071, China

**Keywords:** HIV, genotype, drug resistance genes, Central China

## Abstract

The global distribution of HIV-1 subtypes exhibits significant regional variations, with evolving epidemiological patterns over time. China currently experiences concurrent circulation of multiple HIV-1 subtypes, and the transmission landscape is becoming increasingly complex and diversified. We performed prospective molecular surveillance and drug-resistance profiling of HIV-1 in Wuhan City to delineate the local genotypic structure and to guide antiretroviral therapy. A total of 149 whole blood samples from HIV-1-infected individuals preserved in 2022 at a hospital in Wuhan were selected. Peripheral-blood mononuclear cells (PBMCs) were isolated, total RNA extracted, and the Gag, Pol, and Env regions were amplified by nested RT-PCR and sequenced. The sequencing and phylogenetic tree results revealed that subtype B constituted the predominant clade (73/149, 49.1%), followed by CRF07_BC (20, 13.4%), CRF01_AE (13, 8.7%), CRF55_01B (2, 1.3%), and subtype C (1, 0.7%). Drug resistance mutations were detected in 36 cases, involving 41 mutation sites across 21 distinct types. Resistance profiles included two protease inhibitor-associated mutation sites (2 types), seven nucleoside reverse transcriptase inhibitor (NRTI)-related mutation sites (6 types), and 32 non-nucleoside reverse transcriptase inhibitor (NNRTI)-associated mutation sites (13 types).

## 1. Introduction

According to the UNAIDS 2023 report, as of 2022, 39 million people were living with HIV infection globally, 1.3 million new cases were reported that year, 85.6 million people have been infected with HIV since the beginning of the epidemic, and 40.4 million people have died from AIDS-related illnesses [1]. In 2023, China reported 110,491 cases of HIV/AIDS patients and 34,962 deaths, and as of 31 December 2023, there were 1,289,700 cases of living HIV/AIDS patients and 457,609 deaths in China [2]. As of 2023, Hubei Province has 23,893 people living with HIV/AIDS, ranking 12th in China. A cohort analysis of the HIV-infected population in Hubei Province revealed a general upward trend in new HIV infections between 1995 and 2020 [3].

HIV-1 is categorized into four groups: M, N, O, and P. The distribution of these groups is uneven, with the M group being the most widespread globally, while the N, O, and P groups are primarily localized to certain regions of Africa. As the most globally prevalent strain, HIV-1 group M viruses are further classified into nine subtypes (A–D, F–H, J, and K) and numerous circulating recombinant forms (CRFs) and unique recombinant forms (URFs) [4]. Additionally, the high level of genomic variability has led to further molecular evolutionary subdivisions within some subtypes. For example, subtype A can be divided into subtypes A1–A5 [5]. The diversity of HIV-1 subtypes reflects the adaptive evolution of the virus across different regions and populations. According to the Los Alamos National Laboratory in the United States, 134 CRFs have been identified globally, and recombinant viruses account for 29% of the prevalent HIV-1 strains worldwide, with this proportion continuing to rise [6]. This suggests that recombinant viruses may possess enhanced replication and transmission capabilities [7]. With ongoing viral mutations and increasing global human mobility, the emergence of additional subtypes is likely in the future, presenting greater challenges for HIV-1 monitoring and control. Consequently, recombinant viruses such as CRFs and URFs will be a critical focus of future prevention and control efforts [8].

China has become one of the countries with the most diverse HIV-1 subtypes globally. A total of 18 CRFs were identified. The main prevalent HIV-1 subtypes in China include CRF07_BC, CRF01_AE, CRF08_BC, and subtype B, accounting for 35.5%, 27.6%, 20.1%, and 9.6% of cases, respectively [9]. Among these, CRF07_BC and CRF08_BC were primarily spread among drug-using populations in the northeast and southeast regions, while subtype B predominated among irregular blood donors in the central region. CRF07_BC, CRF01_AE, and CRF55_01B expanded rapidly among MSM [10,11]. The distribution of HIV-1 subtypes has evolved over time. Recent studies [12] indicate that the prevalence of subtypes CRF01_AE and B decreased from 37.3% and 24.1% in 2004–2007 to 29.4% and 7.3% in 2020–2022, respectively. In contrast, CRF07_BC, CRF55_01B, and other CRFs and URFs increased from 24.1%, 0.1%, 0.4%, and 0.9% in 2004–2007 to 40.8%, 3.8%, 3.7%, and 2.8% in 2020–2022, respectively. Overall, China is characterized by the coexistence of multiple HIV-1 subtypes, the frequent emergence of new recombinant strains, and the expansion of epidemic areas, demonstrating increasingly complex and diversified transmission dynamics.

The HIV-1 genome is highly prone to mutation due to its intrinsic mechanism of rapid evolution. The high mutation rate is primarily attributed to the lack of proofreading activity in HIV-1 reverse transcriptase. The mutation rate reaches 3.4 × 10^−5^ per base per replication cycle [13]. Over time, mutant and recombinant strains accumulate in the host, forming a population of closely related but genetically diverse variants, known as quasispecies [14,15,16]. Using phylogenetic tools, researchers can identify specific patterns of HIV genetic diversity, with significant sequence variations observed in structural and regulatory genes across different HIV-1 subtypes. Recombinant strains exhibit a greater capacity for rapid spread compared to single subtypes, and the emergence of CRFs plays a significant role in driving the ongoing HIV/AIDS epidemic [17]. Several studies have demonstrated a strong association between HIV-1 genetic diversity and the rate of disease progression in infected individuals [18]. For instance, some recently infected patients who do not receive timely ART after diagnosis experience accelerated disease progression, rapidly advancing to AIDS.

This study aims to systematically characterize the genotypic distribution and molecular features of HIV-1 in Wuhan.

## 2. Materials and Methods

### 2.1. Specimens

149 whole blood samples were collected from HIV-1-infected patients at Jinyintan Hospital in Wuhan. Peripheral blood mononuclear cells (PBMCs) were isolated, and total RNA was extracted using the Trizol method. Subsequently, cDNA was synthesized through reverse transcription of the extracted RNA.

All patient personal identifiable information was removed at the study’s commencement, and subsequent analyses were based solely on the de-identified data. The Medical Ethics Committee of Wuhan Infectious Disease Hospital (Jinyintan Hospital) reviewed and approved this research protocol (Ethics approval number: KY-2023-38).

### 2.2. HIV-1 RNA Extraction, Nested PCR Amplification, and Sequencing

Nested PCR was performed to amplify specific regions of the HIV-1 gag, pol, and env genes. The first-round PCR reaction mixture was prepared as follows: 1 µL of cDNA template (10 ng/µL), 1 µL of upstream primer (10 µM), 1 µL of downstream primer (10 µM), 10 µL of 2× Es Taq MasterMix, and 7 µL of ddH_2_O, yielding a total reaction volume of 20 µL. The reaction was carried out in a PCR instrument (BIO-RAD, T100™, Hercules, CA, USA) using the following program: initial denaturation at 94 °C for 3 min, followed by 30 cycles of denaturation at 94 °C for 30 s, annealing at 57 °C for 30 s, and extension at 72 °C for 45 s, with a final extension at 72 °C for 10 min. The first-round PCR product was then used as the template for the second-round PCR. The second-round reaction mixture consisted of 1 µL of DNA template (10 ng/µL), 1 µL of upstream primer (10 µM), 1 µL of downstream primer (10 µM), 15 µL of 2× Es Taq MasterMix, and 7 µL of ddH_2_O, resulting in a total reaction volume of 30 µL. The PCR program for the second round was identical to that of the first round. Following PCR amplification, the products were analyzed by electrophoresis on a 1% agarose gel to confirm the expected product sizes. The target bands were excised and purified. The purified PCR products were subsequently sent to DNA sequencing company for analysis. The amplification primers were designed based on the study by Rebecca L. R. Powell et al. [19,20], and their sequences are provided in Table 1.

### 2.3. HIV-1 Sequence Genotype Analysis

The sequencing results were analyzed to determine the genotypes of the gag, pol, and env gene regions based on sequence homology using the HIV-BLAST tool (HIV-BLAST v2.0) available in the HIV Nucleic Acid Sequence Database at Los Alamos National Laboratory, USA. The genotyping results were further validated by constructing a phylogenetic tree using the Neighbor-joining method in MEGA 7 software. The reliability of the Neighbor-joining phylogenetic tree and the topology of the branching patterns were assessed using the Bootstrap test, with a resampling parameter set to 1000 replicates for phylogenetic tree construction.

### 2.4. HIV-1 Genotypic Drug Resistance Analysis

The aligned and manually corrected pol gene sequences were submitted to the Stanford University HIV Drug Resistance Database (https://hivdb.stanford.edu/, accessed on 4 March 2024) for analysis of drug resistance mutation sites using the online Sequence Resistance Site Analysis tool (https://hivdb.stanford.edu/hivdb/by-sequences/, accessed on 19 March 2024).

### 2.5. Statistical Analysis

All survey and experimental data were organized using Microsoft Excel 2016. Statistical analyses were performed using SPSS 19 and GraphPad Prism 8. Categorical data were expressed as rates or proportions, and between-group comparisons of count data were conducted using the chi-square test. Two-sided tests were applied, and differences were considered statistically significant at *p* < 0.05.

## 3. Results

### 3.1. Basic Information of HIV-1-Infected Individuals

In this study, a total of 149 RNA samples were collected from February to May 2022 in Wuhan, comprising 124 males and 25 females, with a male-to-female ratio of 4.96:1 (Table 2). The age distribution of the patients ranged from 22 to 75 years, with a median age of 46 years. The median ages of male and female patients were 43.5 and 59 years, respectively, indicating that the average age of female patients was higher than that of males. Sexual transmission was the predominant mode of transmission, accounting for 83.2% of all cases, including 72 cases of male-to-male transmission and 52 cases of heterosexual transmission. The majority of patients (141/149, 94.6%) were receiving ART. Among these, 120 patients (80.5%) had undetectable or low viral loads (<100 copies/mL), 19 patients (12.8%) had viral loads between 100 and 10,000 copies/mL, and 10 patients (6.7%) had viral loads exceeding 10,000 copies/mL. HIV infection leads to a decline in CD4+ T lymphocyte counts. In this cohort, 92 patients had CD4+ T lymphocyte counts below 500 cells/µL, including 56 patients (37.6%) with counts below 350 cells/µL and 16 patients (10.7%) with counts below 200 cells/µL.

### 3.2. Clinical Characterization of HIV-1-Infected Individuals

**Figure 1:**
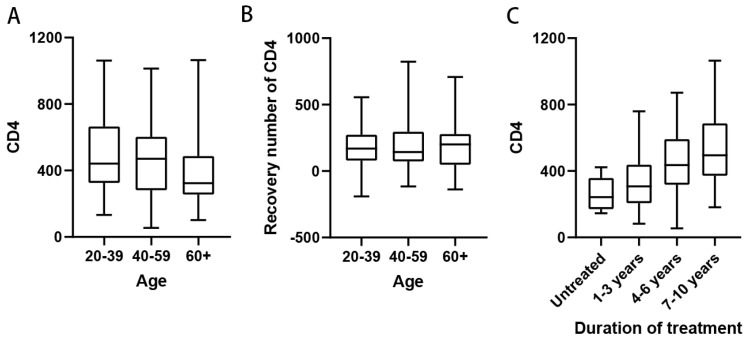
Effect of age and duration of treatment on immune function in HIV-1 patients. (**A**) Peripheral blood CD4+ T cell counts in patients of different ages, patients were 20–39 years old (*n* = 54), 40–59 years old (*n* = 58) and 60 years old and above (*n* = 37); (**B**) Peripheral blood CD4+ T cell counts in patients of different ages who received ART (*n* = 141), patients were 20–39 years old (*n* = 49), 40–59 years old (*n* = 56), and 60 years and older (*n* = 36); (**C**) Number of peripheral blood CD4+ T cells in patients with different times of receiving ART, patients were untreated (*n* = 8), 1–3 years of receiving ART (*n* = 28), 4–6 years (*n* = 54), and 7–10 years (*n* = 37).

Patients were stratified into three age groups: 20–40 years, 40–60 years, and above 60 years. Peripheral blood CD4+ T cell counts were analyzed across these age groups. The results revealed that the median CD4+ T cell counts in patients under 60 years were above 350 cells/µL, whereas the median CD4+ T cell counts in patients above 60 years were below 350 cells/µL (Figure 1A). The immune function status of patients in the older age group was significantly lower than that of the younger age groups (*p* < 0.05). This finding may be attributed to the reduced autoimmunity associated with aging. However, analysis of 103 patients receiving ART showed no significant difference in CD4+ T cell recovery across age groups, indicating that the effect of ART on CD4+ T cell restoration was consistent regardless of age (Figure 1B). Next, we examined the relationship between the duration of ART and peripheral blood CD4+ T cell counts to assess potential correlations. Since none of the patients in this study had received treatment for more than 10 years, they were divided into four groups: untreated, treatment duration of less than 4 years, treatment duration of 4–6 years, and treatment duration of more than 6 years. Peripheral blood CD4+ T cell counts were analyzed in each group. The results demonstrated that the median CD4+ T cell counts in all treatment duration groups exceeded 350 cells/µL. Furthermore, a trend of increasing median CD4+ T cell counts was observed with longer ART duration. In the group receiving ART for more than 6 years, the median CD4+ T cell count surpassed the lower limit of the normal range (500 cells/µL), reaching 512 cells/µL (Figure 1C). This value was significantly higher than that of the short-duration treatment group and the untreated group (*p* < 0.01). These findings indicate that ART contributes to the restoration of peripheral blood CD4+ T cell counts in HIV-1 patients and facilitates immune reconstitution. This further underscores the critical role of early and sustained ART in controlling HIV-1 viral replication and restoring immune function.

### 3.3. Distribution of HIV-1 Genotypes

**Figure 2:**
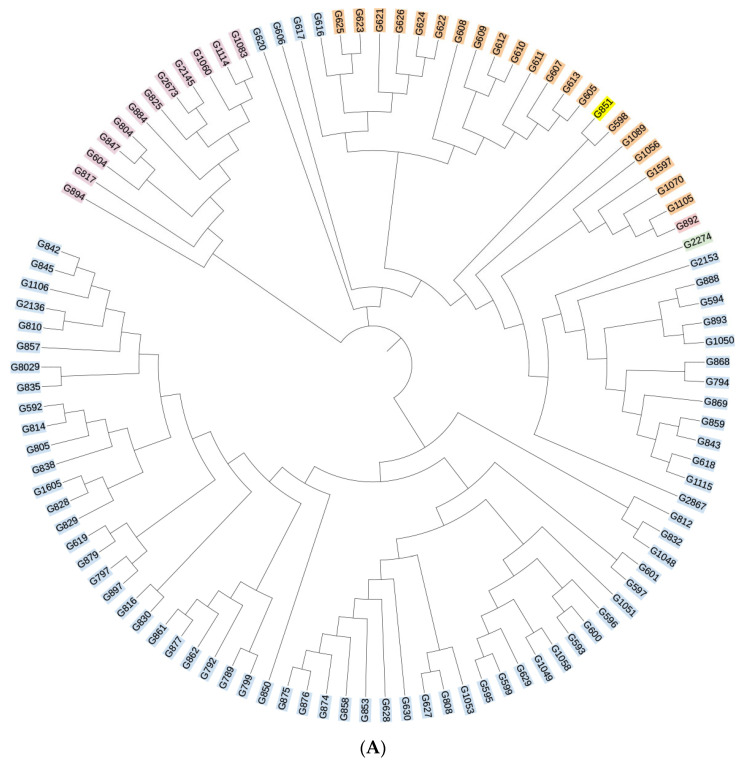

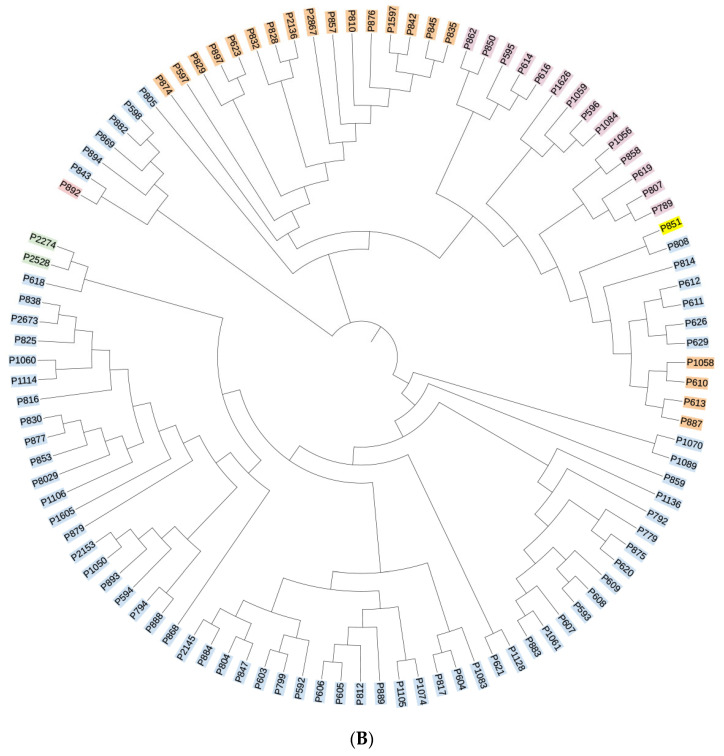

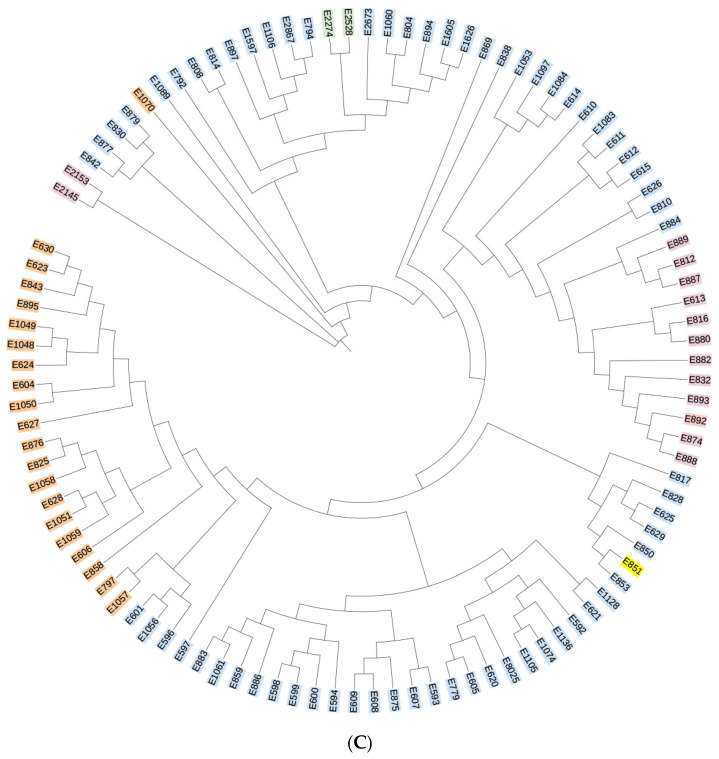
(**A**) Phylogenetic tree of the gag gene region of the HIV-1 virus strain. Blue indicates subtype B, yellow indicates subtype C, orange indicates CRF07_BC, purple indicates CRF01_AE, and green indicates CRF55_01B. (**B**) Phylogenetic tree of the pol gene region of the HIV-1 virus strain. Blue indicates subtype B, yellow indicates subtype C, orange indicates CRF07_BC, purple indicates CRF01_AE, and green indicates CRF55_01B. (**C**) Phylogenetic tree of the env gene region of the HIV-1 virus strain. Blue indicates subtype B, yellow indicates subtype C, orange indicates CRF07_BC, purple indicates CRF01_AE, and green indicates CRF55_01B.

All sequencing results were compared against the HIV databases at Los Alamos National Laboratory, USA. The reference strains are as follows: CRF55_01B-HNCS102056 (JX574661); CRF07_BC-97CN54 (AF286226); CRF01_AE-CM240 (U54771). The analysis revealed that 40 samples could not be definitively classified as recombinant strains due to inconsistencies in the genotyping results across gene fragments. The remaining HIV-1 strains were genotyped into the following subtypes: subtype B, CRF07_BC, CRF01_AE, CRF55_01B, and subtype C. Subtype B was the most prevalent, accounting for 49.1% (73/149) of all samples, followed by CRF07_BC and CRF01_AE at 13.4% (20/149) and 8.7% (13/149), respectively. Additionally, two cases of CRF55_01B and one case of subtype C were identified, with subtype C representing a previously unreported HIV-1 genotype in this region. Phylogenetic trees for the gag, pol, and env genes were constructed using MEGA 4.1 software, and the results are presented in Figure 2A–C. The overall topology of the phylogenetic trees for the three HIV-1 genes was similar, with sample sequences distributed across different genotype clusters.

### 3.4. Trends in HIV-1 Subtype Distribution over Time of Infection

**Figure 3:**
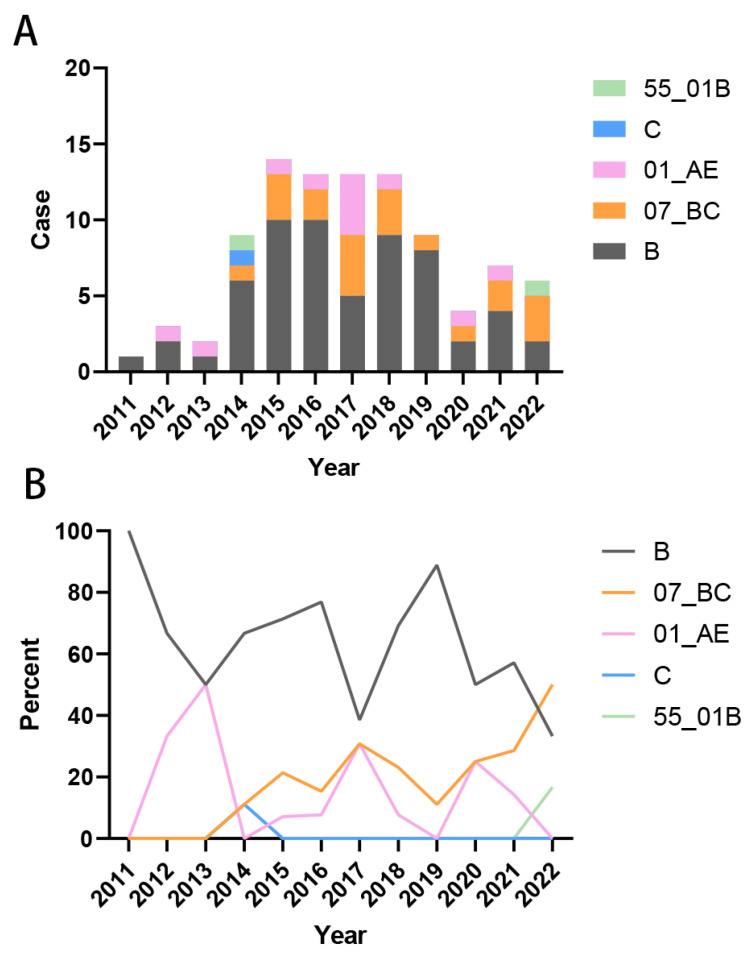
Trends in HIV-1 subtype distribution over time of infection. (**A**) Composition of the number of each subtype of infected strains in 2011–2022; (**B**) Percentage of each subtype of infected strains in 2011–2022.

We compiled the years of initial HIV-1 infection among patients and analyzed the temporal trends in viral subtype distribution. The results indicated that the majority of HIV cases were diagnosed during the period 2014–2019. Prior to 2017, subtype B strains accounted for the largest proportion of infections. From 2011 to 2013, the only circulating strains were subtype B and CRF01_AE (Figure 3A). Beginning in 2014, the diversity of HIV-1 subtypes increased, marked by a gradual decline in the proportion of the previously dominant subtype B and a concurrent rise in the proportion of CRF07_BC infections. By 2022, the proportion of CRF07_BC infections had surpassed that of subtype B (Figure 3B). These findings demonstrate an increasing complexity in the composition of HIV-1 subtypes over time.

### 3.5. Analysis of the Amino Acid Composition of HIV-1 Gene Fragments

The amino acid composition of the three gene fragments (gag, pol, and env) of the isolated strain was analyzed using the HXB2 sequence as the reference. The results are presented in Supplementary Figure S1. Compared to the reference sequence, the amino acid composition of the three gene fragments exhibited varying degrees of divergence. The conserved sites for the gag, pol, and env gene fragments were 27.7% (43/155), 11.4% (57/500), and 11.2% (17/152), respectively. The gag gene fragment showed a higher degree of amino acid conservation, whereas the pol and env gene fragments displayed greater amino acid variability.

### 3.6. Analysis of HIV-1 Drug Resistance Sites

**Figure 4:**
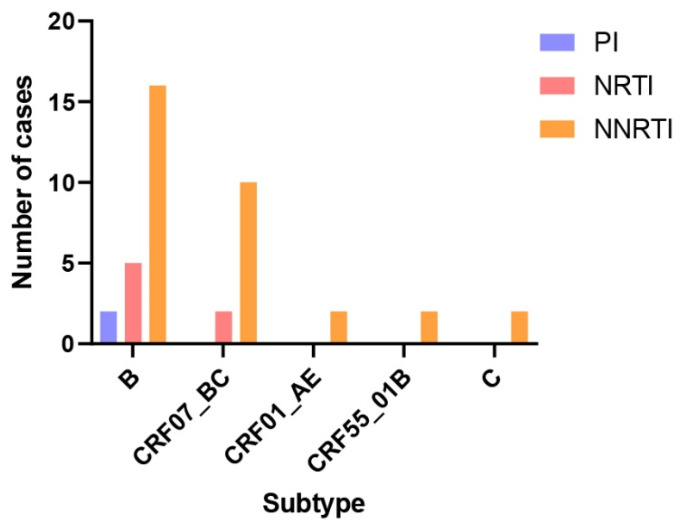
Drug resistance mutation sites in the pol region of different HIV-1 subtype isolates. The number of Pol fragment resistance mutation sites was calculated for each subtype sequence, and a total of 23 resistance mutation sites were found in subtype B (2 PI-resistant sites, 5 NRTI-resistant sites, and 16 NNRTI-resistant sites), a total of 12 resistance mutation sites were found in subtype CRF07_BC (2 NRTI-resistant sites, and 10 NNRTI-resistant sites), and CRF01_AE, CRF55_01B and C subtypes each identified 2 NNRTI resistance loci.

The pol gene sequences of the isolated strains were submitted to the Stanford University HIV Drug Resistance Database for drug resistance mutation analysis. A total of 36 cases exhibited drug resistance-related mutations, encompassing 41 mutation sites and 21 mutation types. These included 2 protease inhibitor (PI) resistance mutation sites with 2 mutation types, 7 nucleoside reverse transcriptase inhibitor (NRTI) resistance mutation sites with 6 mutation types, and 32 non-nucleoside reverse transcriptase inhibitor (NNRTI) resistance mutation sites with 13 mutation types (Table 3, Figure 4). The V179E mutation was detected in 6 isolates, representing the most frequent resistance mutation site identified in this study. Among seven ART-treated patients with viral loads exceeding 100 copies/mL, resistance mutations were detected in all cases: one patient harbored NRTI resistance mutations, while six patients exhibited NNRTI resistance mutations. In our study, the V179E resistance mutation was detected in two patients infected with the CRF55_01B strain. One of these patients received ART for 7 years, starting with an initial regimen of EFV + 3TC + AZT, which was later switched to 3TC + AZT + LPV/r (the exact timing of the switch was unknown). At the time of sampling, this patient had a viral load of 4210 copies/mL and a CD4+ T cell count of 260 cells/µL. Another patient, infected with a subtype C strain, harbored the M230I and P236L resistance mutations. This patient had received ART for 8 years, beginning with EFV + TDF + 3TC and later switching to BIC/FTC/TAF (the specific timing of the switch was unknown). At sampling, the viral load was 324 copies/mL, and the CD4+ T cell count was 48 cells/µL. Additionally, four specimens in this study carried more than one resistance mutation site. Among these, three infected patients exhibited clinical drug resistance, with viral loads exceeding 200 copies/mL at the time of sampling.

## 4. Discussion

In this study, the majority of ART-treated patients achieved viral loads below the minimum detection threshold (85.7%, 120/140), and over 60% of patients had peripheral blood CD4+ T lymphocyte counts of 350 cells/µL or higher (64.3%, 90/140). These findings underscore the importance of ART adherence in suppressing viral replication, reducing viral loads, minimizing immune cell damage, promoting immune reconstitution, and delaying AIDS progression. Additionally, age was found to influence ART treatment efficacy. Among the 141 ART-treated patients in this study, 120 (85.1%) achieved treatment success (viral load < 100 copies/mL or undetectable). However, the probability of treatment failure (viral load > 100 copies/mL) varied across age groups: 12.2% (6/49) in patients aged 20–40 years, 16.1% (9/56) in patients aged 40–60 years, and 16.7% (6/36) in patients aged 60 years and above. The reduced treatment efficacy in older patients may be attributed to age-related declines in physiological function, the presence of comorbidities, and relatively weaker immune responses. These factors may prolong the time required to control HIV-1 viral loads and slow immune reconstitution, resulting in lower post-treatment immune recovery levels compared to younger patients. Furthermore, older individuals are more prone to comorbid conditions, declines in cardiovascular, hepatic, and renal function, and slower drug metabolism, increasing the risk of drug accumulation and adverse reactions.

In the early stages of the HIV-1 epidemic in China, transmission primarily occurred through bloodborne routes, including intravenous drug use and unregulated blood donation. By the end of the last century, unregulated blood donation had facilitated the widespread dissemination of subtype B in the central region, establishing it as the dominant strain in provinces such as Henan and Hubei [21]. In this study, subtype B remained the most prevalent among the genotyped strains, although its proportion has declined in recent years. Concurrently, the proportion of infections caused by recombinant strains, such as CRF07_BC and CRF01_AE, has increased. This trend aligns with the growing complexity of HIV-1 strain prevalence in China [12]. Over time, the primary mode of transmission has shifted from bloodborne routes to sexual transmission, with a notable rise in infections among MSM. Sexual transmission has now become the predominant mode of HIV-1 transmission in China. Studies have shown that the co-circulation of CRF01_AE and subtype B among MSM facilitates the emergence of recombinant strains such as CRF55_01B, which is currently the fifth most prevalent strain in China [22,23,24]. In this study, 124 patients (83.2%) were infected through sexual transmission, including 72 cases of male-to-male transmission and 52 cases of heterosexual transmission. The proportion of MSM transmission has been steadily increasing in recent years, consistent with findings from other studies in the literature.

Gene polymorphisms significantly influence the viral infection process. In this study, amino acid composition analysis of HIV-1 isolates revealed greater variability in the pol and env gene regions compared to the gag gene region. During viral entry into host cells, the polymorphism of the Env protein determines its binding affinity and specificity to host cell surface receptors, primarily CD4 and co-receptors CCR5 or CXCR4. The structural variability of the Env protein enables it to evade initial recognition by the host immune system, increasing the likelihood of successful viral entry and facilitating the establishment of infection [25,26]. HIV-1 genetic polymorphisms also play a central role in disease pathogenesis. Once infection is established, viral strains with different genetic profiles exhibit selective advantages in infecting specific tissues and organs. For example, mutations in the V3 loop region of the env gene enhance the virus’s ability to cross the blood–brain barrier, leading to associated neurological lesions such as gray and white matter abnormalities, astrocyte proliferation, and demyelination in periventricular and central white matter [27,28,29]. These changes can progress to AIDS dementia syndrome. Furthermore, genetic variability in HIV-1 promotes immune escape, making it challenging for the host immune system to mount a sustained and effective immune response. On one hand, cytotoxic T lymphocyte (CTL)-recognized viral antigenic epitopes are altered due to genetic variation, preventing their effective clearance [30,31]. On the other hand, neutralizing antibodies struggle to target the continuously evolving viral envelope. These mechanisms not only facilitate persistent viral replication but also exacerbate chronic immune activation and depletion, ultimately driving disease progression to AIDS [32].

The polymorphism of the gag gene significantly impacts the efficiency of viral particle assembly. Variations in the gag gene sequence can lead to subtle structural changes in the encoded core proteins, disrupting interactions between these proteins. Consequently, the core proteins may fail to assemble into a complete and stable viral core through the normal process. Mutations in the corresponding protein structures can compromise the formation of stable viral particles, thereby impairing viral infectivity and replication [33,34]. Lucile Larrouy et al. demonstrated that gag mutations influence the virologic response to dual-boosted protease inhibitor combinations in treatment-naïve patients with HIV [35]. They also found that polymorphisms in the protease and gag cleavage sites are associated with poor virologic responses to protease inhibitor-based therapies [36,37,38]. Studies have shown that in some long-term non-progressors (LTNPs)—a subset of patients exhibiting slow disease progression after HIV-1 infection—viral isolates carry specific gag gene mutations. These mutations result in slower but more compact viral particle assembly, which may help the virus evade early detection by the host immune system and facilitate the establishment of infection [39]. Additionally, an analysis of HIV-1 prevalent strains in a high-prevalence region in Africa [40] revealed that gag gene subtypes in this region differ significantly from those in other regions. This genetic polymorphism contributes to a unique transmission pattern characterized by insidious early-stage symptoms and a gradual but persistent increase in viral load, posing significant challenges for early diagnosis and epidemic control. Furthermore, studies on mother-to-child transmission (MTCT) [41,42] have identified that certain strains carrying specific gag polymorphisms are more likely to cross the placental barrier and infect the fetus. Even when maternal viral loads are relatively low, the risk of MTCT remains elevated.

In this study, the pol region genes were analyzed, revealing that 36 patients (24.2%) were infected with strains harboring a total of 41 drug resistance mutation sites. The majority of these mutations were associated with non-nucleoside reverse transcriptase inhibitors (NNRTIs) (32/41, 78.0%), followed by nucleoside reverse transcriptase inhibitors (NRTIs) (7/41, 17.1%) and protease inhibitors (PIs) (2/41, 4.9%). Among these mutations, the V179E mutation was detected in six cases involving subtype B, CRF07_BC, and CRF55_01B strains. The V179E mutation is a nonpolymorphic mutation that may confer varying degrees of resistance to NNRTIs. Specifically, it has been associated with resistance to efavirenz (EFV), while its impact on nevirapine (NVP) resistance appears to be relatively weak [43,44]. Studies have shown that the V179E mutation is prevalent in most CRF55_01B strains [45], a finding corroborated by its detection in both CRF55_01B strains in this study. Therefore, close monitoring of the epidemiological characteristics of CRF55_01B strains and the clinical indicators of infected patients is essential. Due to the high mutation rate of HIV-1 and the selective pressure exerted by antiretroviral drugs, mutant and wild-type strains undergo complex dynamic changes, leading to genetic polymorphism and variability. This process further drives the emergence of drug-resistant viruses and the development of acquired drug resistance [45,46]. Drug resistance significantly undermines the efficacy of highly active antiretroviral therapy (HAART) in reducing and controlling viral loads, adversely affecting disease progression and outcomes [47]. Moreover, drug-resistant HIV-1 strains can continue to spread, contributing to the dissemination of drug resistance. Most commonly used antiretroviral drugs target key viral enzymes or proteins, such as reverse transcriptase inhibitors and protease inhibitors. In recent years, additional classes of drugs, including integrase strand transfer inhibitors (INSTIs), fusion inhibitors (FIs), and CCR5 antagonists, have been introduced into clinical practice [48,49,50,51]. However, the rapid mutation rate of viral genes has led to the frequent emergence of drug-resistant variants. Amino acid changes at drug-binding sites reduce drug affinity and inhibitory activity, significantly increasing the risk of treatment failure. This underscores the need for ongoing research and development of novel drugs and the optimization of combination therapies to address the growing complexity and variability of viral genotypes.

This study is exploratory in nature and has limitations due to its small sample size and potential data bias arising from individual patient factors. Further research is needed to control for confounding variables and expand the sample size to strengthen the validity of the findings.

## 5. Conclusions

The prevalence of HIV-1 strains in Wuhan, Hubei Province, has shifted from a predominance of subtype B to a mixture of multiple subtypes and recombinant forms. The complexity of subtype composition among prevalent strains is expected to increase further in the future. The clinical immune function of HIV-1-infected patients is primarily associated with age and the duration of ART, with minimal influence from the genotype of the infecting strain. However, viral gene polymorphisms, particularly mutations in the pol region, significantly impact drug resistance.

## Figures and Tables

**Table 1:** Primers for nested PCR amplification of HIV-1 genes.

Procedure	Name	Sequences (5′–3′)	Position
gag	GexF	GCTGAAGCGCGCACGGCAAGAG	705–726
	GexR	AAGGGTACTAGTAGTTCCTGCTATG	1518–1494
	GinF	TTTGACTAGCGGAGGCTAGA	761–780
	GinR	GCCTGATGTACCATTTGCCC	1226–1207
pol	PexF	GTAAAAAATTGGATGACAGAAACCTTG	1726–1752
	PexR	CTGTATTTCTGCTATTAAGTCTTTTGATGGG	3509–3539
	PinF	CATAGCCAAAAATTGCAGGGCCCCTAGRAAAAAG	1989–2022
	PinR	AATACACTCCATGTACCGGTGTTTTTAAAATCTCYC	3469–3504
env	EexF	TCTTAGGAGCAGCAGGAAGCACTATGGG	7789–7816
	EexR	AACGACAAAGGTGAGTATCCCTGCCTAA	8347–8371
	EinF	ACAATTATTGTCTGGTATAGTGCAACAGCA	7850–7879
	EinR	TTAAACCTATCAAGCCTCCTACTATCATTA	8281–8310

**Table 2:** Basic characteristics of the study population.

Variable	Number of Cases	Constituent Ratio (%)
Total	149	100
Gender		
Male	124	83.2
Female	25	16.8
Age		
20–<40	54	36.2
40–<60	58	39.0
≥60	37	24.8
Infection Routes		
Sexual contact	124	83.2
homosexual	72	48.3
heterosexual	52	34.9
drug injection	1	0.7
Unknown	24	16.1
Viral load		
TND/<100	120	80.5
100–<10,000	19	12.8
≥10,000	10	6.7
CD4		
<350	56	37.6
≥350	92	61.7
Null	1	0.7

**Table 3:** Drug resistance mutation sites in the pol region of HIV-1 isolates.

Subtype	Drug-Resistant Mutation		
	PIs	NRTIs	NNRTIs
B	T74P; L33F	F116Y; Y115F; K219Q; T215Y	V179E; M230I; P236L; V108I; F227I; E138G; M230L; K238T
CRF07_BC	-	D67E; T215I	V106L; V179E; M230I; L100V; K238N; K238T
CRF01_AE	-	-	K103T; F227I
CRF55_01B	-	-	V179E
**C**	-	-	M230I; P236L

## Data Availability

The data presented in this study are available on request from the corresponding author due to ethical reasons.

## Supplementary Materials

The following supporting information can be downloaded at: https://www.mdpi.com/article/10.3390/v18010055/s1, Figure S1: Amino acid composition of the gag, pol, gene fragment of the HIV-1 virus strain.
