# Supplementary material for: Molecular Epidemiological Surveillance of HIV-1 Genotypes and Drug Resistance Profiles in Wuhan, Central China
Source: Viruses. 2025 Dec 30;18(1):55. doi: 10.3390/v18010055 (PMC12846347; doi:10.3390/v18010055)
Supplement: Supplementary file 1 [file viruses-18-00055-s001.zip › viruses-4020443-supplementary.pdf]

Supplementary files

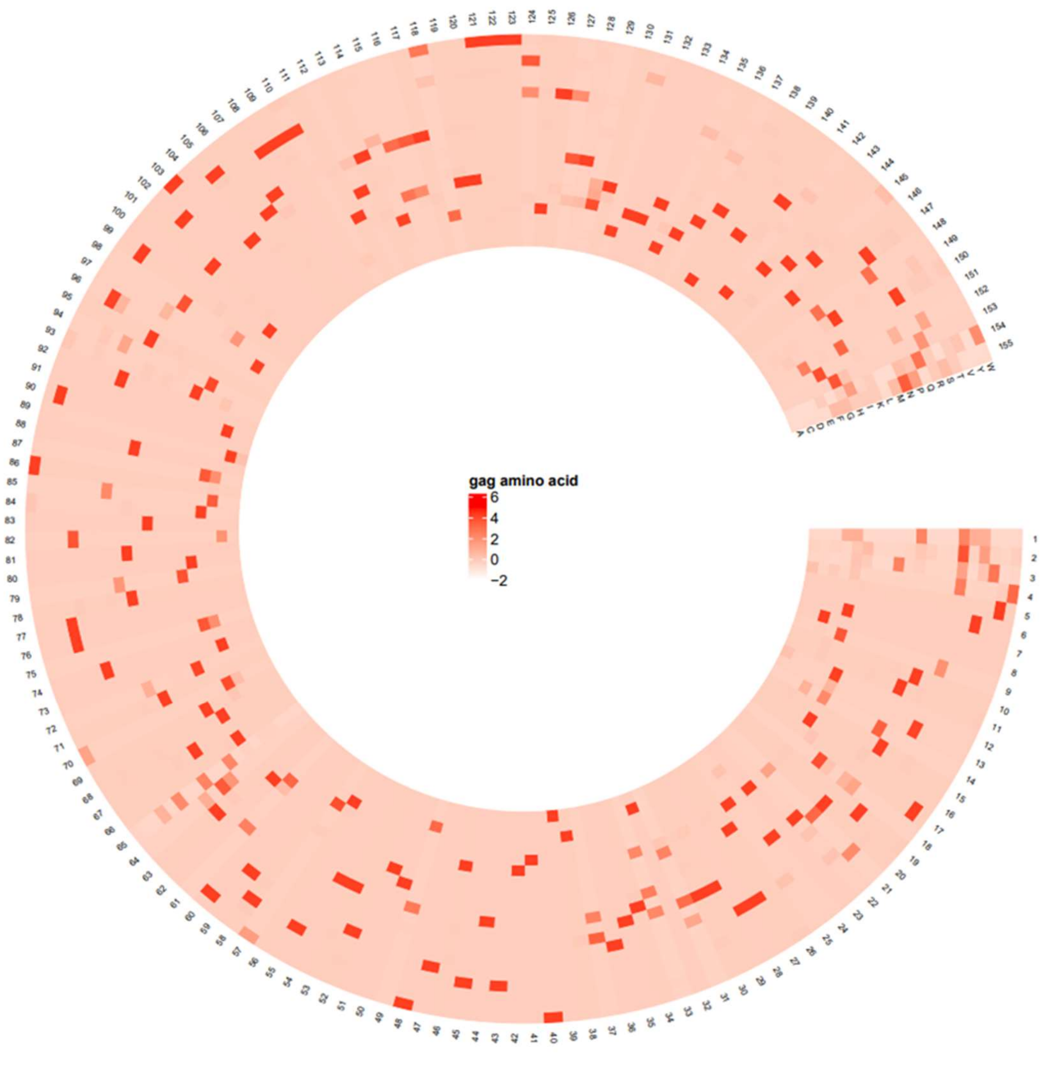

(**A**)

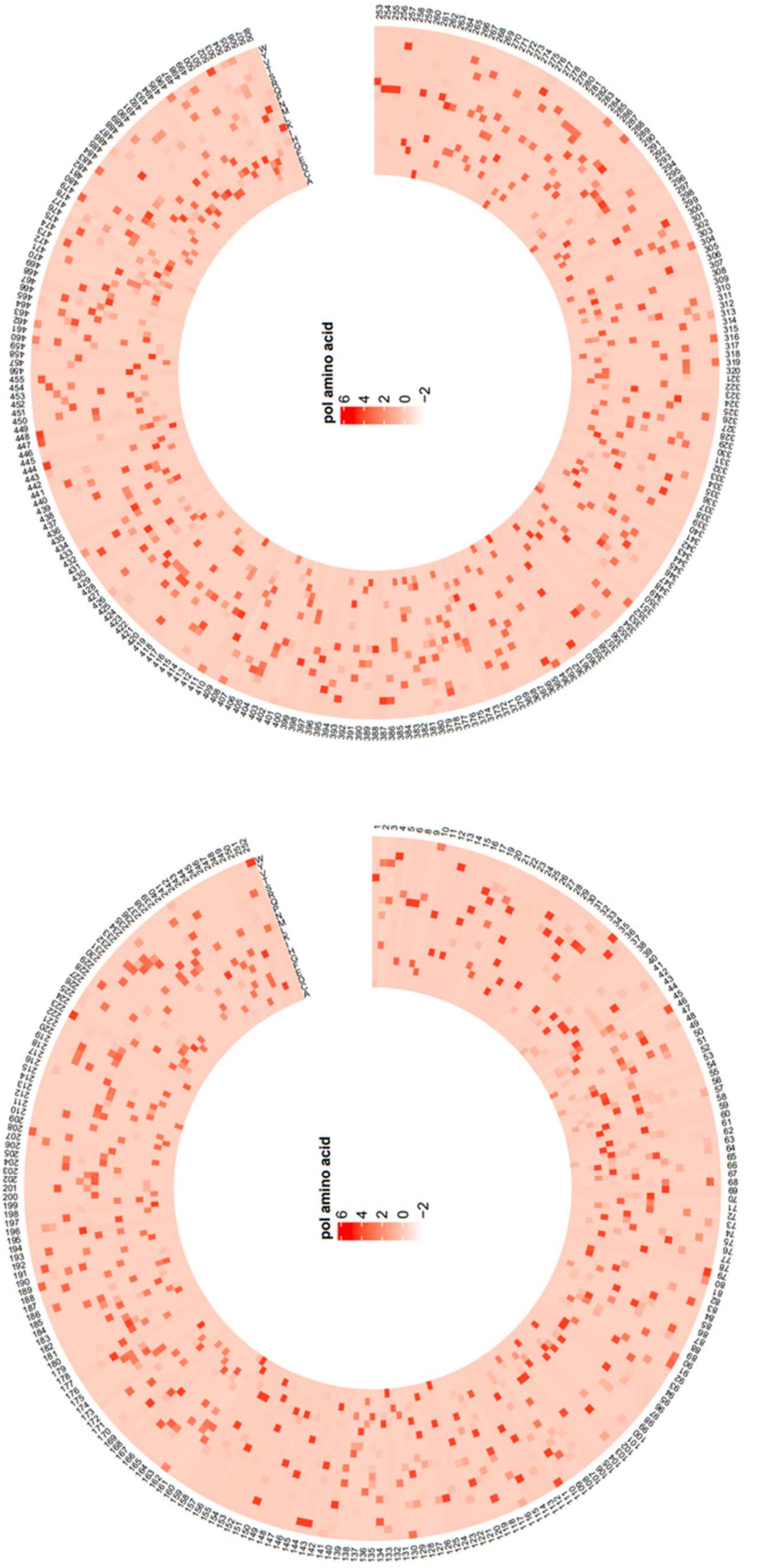

(**B**)

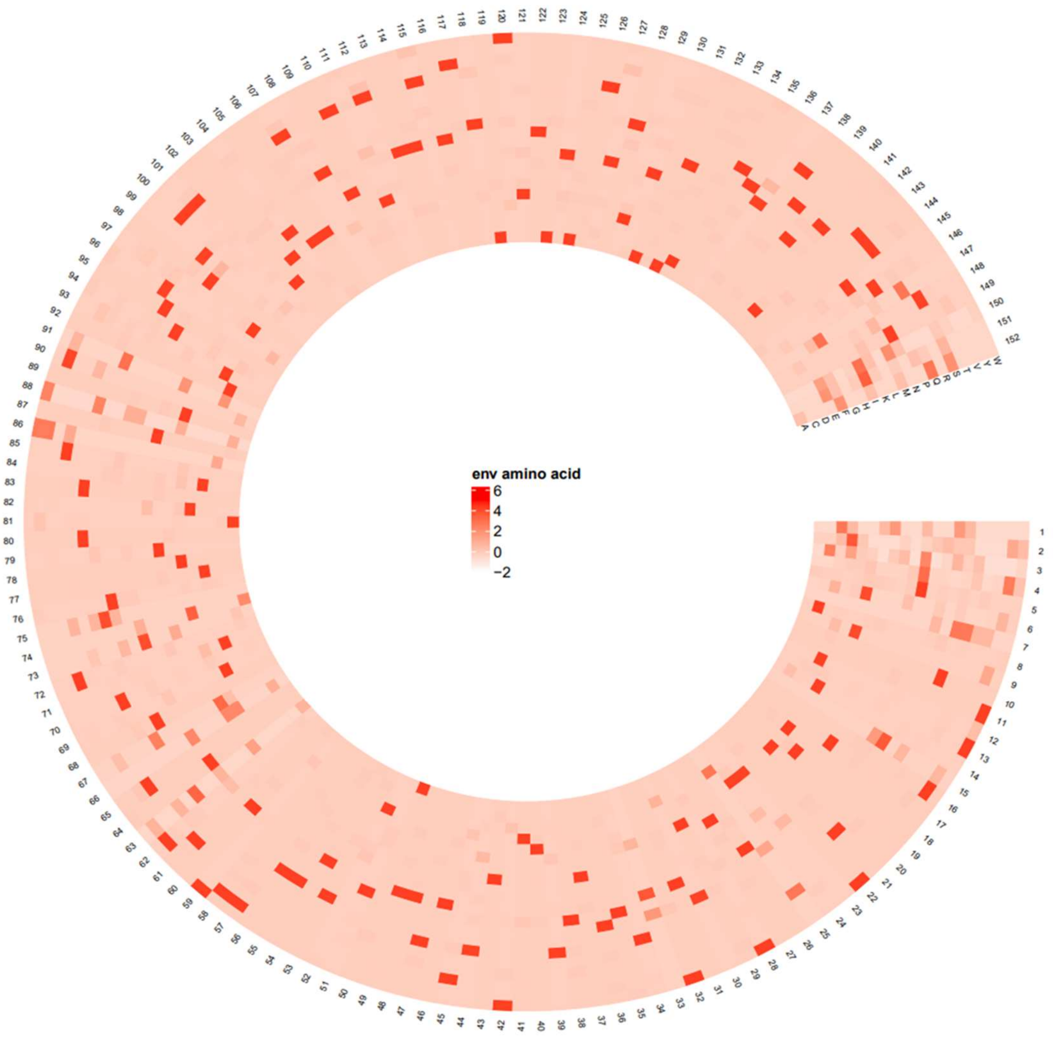

(**C**)

Supplementary Figure S1. Amino acid composition of the (A) gag, (B) pol, (C) env gene fragment of the HIV-1 virus strain
